# Respiratory symptoms increase health care consumption and affect everyday life – a cross-sectional population-based study from Finland, Estonia, and Sweden

**DOI:** 10.3402/ecrj.v3.31024

**Published:** 2016-05-27

**Authors:** Malin Axelsson, Anne Lindberg, Annette Kainu, Eva Rönmark, Sven-Arne Jansson

**Affiliations:** 1 Department of Care Science, Faculty of Health and Society, Malmö University, Malmö, Sweden; 2 Department of Public Health and Clinical Medicine, Division of Medicine, The OLIN Unit, Umeå University, Umeå, Sweden; 3 HUCH Heart and Lung Center, Helsinki University Hospital and University of Helsinki, Helsinki, Finland; 4 Department of Public Health and Clinical Medicine, Occupational and Environmental Medicine, The OLIN Unit, Umeå University, Umeå, Sweden

**Keywords:** epidemiology, daily life, health care utilisation, respiratory symptoms, risk factors, structured interviews

## Abstract

**Background:**

Even though respiratory symptoms are common in the adult population, there is limited research describing their impact on everyday life and association with health care consumption.

**Aim:**

The main objective of this population-based study was to estimate and compare the prevalence of respiratory symptoms among adults in Finland, Estonia, and Sweden in relation to health care consumption and to identify factors influencing health care consumption. A secondary aim was to assess to which extent the presence of respiratory symptoms affect everyday life.

**Method:**

In the population-based FinEsS studies consisting of random samples of subjects aged 20 to 69 years from Finland (*n*=1,337), Estonia (*n*=1,346), and Sweden (*n*=1,953), data on demographics, respiratory health, and health care consumption were collected by structured interviews. Prevalence was compared and multiple logistic regression analyses were performed.

**Results:**

Respiratory symptoms were significantly more common in Finland (66.0%) and Estonia (65.2%) than in Sweden (54.1%). Among subjects with respiratory symptoms, the proportion reporting outpatient care during the past year was fairly similar in the three countries, while specialist consultations were more common in Finland (19.1%), and hospitalisations more common in Estonia (15.0%). Finnish and Estonian residency, female sex, and BMI>25 increased the risk for outpatient care consumption. Wheeze and attacks of shortness of breath in the past 12 months, recurrent sputum production, and cough were associated with an increased risk for health care consumption. Increasing number of respiratory symptoms increased the risk for consuming health care. A larger proportion of subjects in Estonia and Sweden experienced their everyday life being affected by respiratory symptoms compared with subjects in Finland.

**Conclusion:**

Respiratory symptoms are common in Finland, Estonia, and Sweden and contribute to a negative impact on everyday life as well as increased health care consumption. The observed differences in health care consumption between countries are probably related to national differences in health care structure.

Respiratory symptoms constitute common health problems in the adult population (1–5). For example, around 20% of the population in the US report at least one chronic respiratory symptom (4), and in the BOLD study, the prevalence of dyspnoea has been estimated to be 27% based on populations from 15 countries (6). Population-based studies in the capitals of Sweden and Finland have shown that the prevalence of respiratory symptoms have remained on a similar level between 1996 and 2007 (7, 8), whilst a decreasing trend was observed in northern Sweden (9). Respiratory symptoms have been associated with increasing age (1, 10), family history of obstructive airway disease (1, 11), urban living (10), and smoking (1, 3, 11). Sex differences have also been observed; females report dyspnoea more often than males (5, 6, 10) while males more often report respiratory symptoms such as cough and phlegm (10). In addition, low socio-economic status has been identified as a risk factor for respiratory symptoms regardless of smoking, age, sex, and family history of asthma (12). Environmental factors such as biomass smoke (13) and various occupational exposures have also been identified as risk factors for respiratory symptoms (3, 14–16).

Even though respiratory symptoms are common among adults (1–5), there is limited research describing the impact of respiratory symptoms on everyday life and the association between respiratory symptoms and health care consumption. Previous studies on health care consumption have primarily focused on the economic burden of obstructive lung diseases on the health care system (17–21) and not on symptoms. The same applies for health-related quality of life (HRQL), which is most often described in relation to a specific obstructive lung disease and not to respiratory symptoms. However, it has been reported that subjects with respiratory symptoms have both poorer physical and mental HRQL compared with asymptomatic subjects (22), and increasing burden of respiratory symptoms, assessed as number of respiratory symptoms, have been shown to be associated with deteriorating HRQL (5).

Our hypothesis is that individuals seek health care because of the discomfort associated with respiratory symptoms; it is primarily the experience of respiratory symptoms and not a given diagnosis that affects everyday life and influences health care consumption. Previous studies have hardly addressed these questions.

The main objective of this population-based study was to estimate and compare the prevalence of respiratory symptoms among adults in Finland, Estonia, and Sweden in relation to health care consumption and to identify factors influencing health care consumption. A secondary aim was to assess to which extent the presence of respiratory symptoms affect everyday life.

## Materials and methods

### Study population

Initially, the sample was derived from a postal questionnaire study conducted among randomly selected participants from the general population aged 20–69 years in eight centres in Finland, Estonia, and Sweden in 1996. From the responders to the postal questionnaire at each centre, a random sample of subjects was invited for examination including a structured interview, in which 4,636 subjects participated. This article is based on data from the structured interviews performed around the turn of the millennium. The study was approved by regional ethical boards in each of the three countries.

### Data collection

Data were collected through structured interviews using the FinEsS (Finland, Estonia, Sweden) interview questionnaire, which is based on the Obstructive Lung Disease in Northern Sweden Studies (OLIN) questionnaire (23), which in turn was developed from the British Medical Research Council questionnaire (24). The FinEsS questionnaire was translated from English to the native languages in each country, and it has been used in several epidemiological studies (8, 12, 25–27). The questionnaire included questions regarding respiratory symptoms, smoking habits, working status, health care consumption, and the extent to which respiratory symptoms affect everyday life. In addition, data on height and weight were recorded.

### Definitions

Definitions of the variables used in the analyses

Any respiratory symptoms during the past 12 months: yes to any one of the definitions 1–5 below.

1. Recurrent morning cough: ‘Usually cough in the morning’.
2. Recurrent day- and night-time cough: ‘Usually cough during other times of the day or at night’.
3. Recurrent sputum production: ‘Usually brings up phlegm from your chest when coughing or hawking’.
4. Wheeze in the past 12 months: ‘Wheezing or whistling in your chest at any time in the last 12 months’.
5. Attacks of shortness of breath in the past 12 months: ‘Had any attacks of shortness of breath or breathlessness in the last 12 months’.

Based on the respiratory symptoms above, a cumulative symptom score ranging from 0 to 5 symptoms was created, showing how many symptoms the subjects reported.

1. Outpatient care during the past 12 months: ‘Have you consulted a physician due to breathing problems or cough or phlegm during the past 12 months?’
2. Regular outpatient care: ‘Do you regularly see a physician due to your breathing problems?’
3. Specialist consultation: ‘Have you, due to breathing problems or cough or phlegm, consulted a specialist in respiratory medicine or allergology?’
4. Hospitalisations: ‘Have you been treated as an in-patient in a hospital due to breathing problems?’

Working status was classified as working, old age pension, or not working. Not working included subjects classified as disability pension, job seekers, and other.

Subjects rated to which extent their respiratory symptoms affected their everyday life among the following options: not at all, slightly, sometimes moderately, moderately, or severely. A dichotomous variable – impact/no impact on everyday life – was constructed. Impact on everyday life consisted of the response alternatives, namely sometimes moderately/moderately/severely, and no impact of everyday life consisted of the response alternatives not at all/slightly.

### Statistical analysis

Statistical analyses were performed with the IBM Statistical Package for the Social Sciences (IBM Corp. Released 2012. IBM SPSS Statistics for Windows, Version 21.0. Armonk, NY: IBM Corp.). Descriptive statistics, that is, percentages and frequencies were used to describe the study sample and proportions of health care consumption. The Chi-square test was used to test differences in proportion between groups. Mantel–Haenszel test was used to test trends in variables with more than two response alternatives. Multiple logistic regression analyses expressed as odds ratio (OR) with 95% CI were used to identify risk factors for health care consumption. Differences between groups were regarded as significant if *p*<0.05.

## Results

### Respiratory symptoms by country and sex

Basic characteristics of the study population are shown in Table 1. Prevalence estimations of respiratory symptoms in each country and among males and females are presented in Table 2. In the total sample, the prevalence of any respiratory symptoms during the past 12 months was significantly more common in Finland (66.0%) as well as in Estonia (65.2%), when compared with in Sweden (54.1%), and the same prevalence pattern was seen in both males and females. The prevalence of each investigated symptom varied between the countries. The prevalence of recurrent morning cough was significantly higher in Sweden compared with in Finland and Estonia both in the total sample and in females. In males, the prevalence of recurrent morning cough was higher in both Estonia and Sweden than in Finland. In the total sample, recurrent sputum production was more common in Finland and Estonia than in Sweden, and the same pattern was seen in both males and females. The prevalence of wheeze in the past 12 months was higher in Sweden than in Finland. In males, the prevalence was higher in Estonia, and in females the prevalence was higher in Sweden (Table 2).

**Table 1:** Basic characteristics of the study population by country and sex

	All subjects			Males			Females		
			
	Finland (*n*=1,337)	Estonia (*n*=1,346)	Sweden (*n*=1,953)	Finland (*n*=597)	Estonia (*n*=612)	Sweden (*n*=971)	Finland (*n*=740)	Estonia (*n*=734)	Sweden (*n*=982)
Age, mean (SD)	44.8 (13.1)	43.8 (12.9)	45.7 (13.4)	45.3 (13.2)	44.1 (13.2)	46.2 (13.4)	44.5 (12.9)	43.5 (12.8)	45.3 (13.4)
Male, *n* (%)	597 (44.7)	612 (45.5)	971 (49.7)						
BMI	26.12 (4.5)	25.87 (4.5)	25.45 (3.9)	26.45 (3.8)	25.90 (4.1)	25.38 (5.3)	25.91 (5.0)	25.85 (4.8)	24.22 (5.8)
<20, *n* (%)	53 (4.0)	60 (5.0)	87 (4.6)	9 (1.5)	15 (2.7)	18 (1.9)	44 (5.9)	45 (6.9)	69 (7.3)
20–25, *n* (%)	569 (42.6)	521 (43.6)	879 (46.4)	225 (37.8)	248 (45.3)	392 (41.6)	344 (46.5)	273 (42.1)	487 (51.3)
25–30, *n* (%)	481 (36.0)	425 (35.5)	694 (36.7)	268 (45.0)	213 (38.9)	406 (43.1)	213 (28.8)	212 (32.7)	288 (30.3)
>30, *n* (%)	233 (17.4)	190 (15.9)	233 (12.3)	94 (15.8)	72 (13.1)	127 (13.5)	139 (18.8)	118 (18.2)	106 (11.2)
Smoking status									
Current smokers, *n* (%)	378 (28.3)	472 (35.1)	458 (23.5)	195 (32.7)	304 (49.7)	223 (23.0)	183 (24.7)	168 (22.9)	235 (23.9)
Ex-smokers, *n* (%)	380 (28.4)	234 (17.4)	579 (29.6)	216 (36.2)	156 (25.5)	313 (32.2)	164 (22.2)	78 (10.6)	266 (27.1)
Non-smokers, *n* (%)	579 (43.3)	638 (47.4)	908 (46.5)	186 (31.2)	151 (24.7)	433 (44.6)	393 (53.1)	487 (66.4)	475 (48.3)
Working status									
Studying, *n* (%)	38 (2.8)	18 (1.3)	70 (3.6)	15 (2.5)	5 (0.8)	19 (2.0)	23 (3.1)	13 (1.8)	51 (5.2)
Working, *n* (%)	809 (60.5)	923 (68.6)	1312 (67.2)	378 (63.3)	442 (72.2)	670 (69.6)	431 (58.2)	481 (65.5)	642 (65.6)
Job seeker, *n* (%)	109 (8.2)	91 (6.8)	89 (4.6)	44 (7.4)	45 (7.4)	47 (4.9)	65 (8.8)	46 (6.3)	42 (4.3)
Disability pension, *n* (%)	134 (10.1)	81 (6.1)	205 (10.5)	48 (8.1)	45 (7.4)	106 (11.0)	86 (11.6)	36 (4.9)	99 (10.1)
Old age pension, *n* (%)	162 (12.1)	181 (13.4)	209 (10.7)	82 (13.7)	62 (10.1)	102 (10.6)	80 (10.8)	119 (16.2)	107 (10.9)
Other, *n* (%)	85 (6.4)	52 (3.8)	68 (3.5)	30 (5.0)	13 (2.1)	19 (2.0)	55 (7.4)	39 (5.4)	37 (3.7)

SD=Standard deviation, BMI=body mass index.

**Table 2:** Comparing prevalence (%) of respiratory symptoms between countries, in the total study population and stratified by sex

	Country				Males				Females			
			
	Finland	Estonia	Sweden	*p*^#^	Finland	Estonia	Sweden	*p*^#^	Finland	Estonia	Sweden	*p*^#^
Any respiratory symptoms during the past 12 months	66.0	65.2	54.1	F/E^ns^ F/S*** E/S***	66.8	67.8	52.9	F/E^ns^ F/S*** E/S***	65.4	63.1	55.3	F/E^ns^ F/S*** E/S***
Recurrent morning cough	23.2	24.1	29.2	F/E^ns^ F/S*** E/S***	24.5	29.6	29.7	F/E* F/S* E/S^ns^	22.2	19.5	28.8	F/E^ns^ F/S** E/S***
Recurrent day- or night-time cough	26.6	25.0	27.9	F/E^ns^ F/S^ns^ E/S^ns^	24.8	24.2	26.2	F/E^ns^ F/S^ns^ E/S^ns^	28.1	25.7	29.6	F/E^ns^ F/S^ns^ E/S^ns^
Recurrent sputum production: • Now and then • Often	39.0 11.1	41.3 8.6	19.6 10.1	F/E^ns^ F/S*** E/S***	40.9 12.4	43.3 11.1	18.7 12.2	F/E^ns^ F/S*** E/S***	37.6 10.0	39.6 6.5	20.4 8.0	F/E^ns^ F/S*** E/S***
Wheeze in the past 12 months	23.7	27.0	27.8	F/E^ns^ F/S** E/S^ns^	24.8	32.2	28.3	F/E** F/S^ns^ E/S^ns^	22.8	22.6	27.3	F/E^ns^ F/S* E/S*
Attacks of shortness of breath in the past 12 months	14.8	13.8	15.3	F/E^ns^ F/S^ns^ E/S^ns^	12.9	11.1	14.0	F/E^ns^ F/S^ns^ E/S^ns^	16.4	16.1	16.6	F/E^ns^ F/S^ns^ E/S^ns^

F=Finland, E=Estonia, S=Sweden, ns=non-significant. *p*>0.05

* *p*<0.05

** *p*<0.01

*** *p*<0.001, *p*-values based on Chi-square test and Mantel–Haenszel test in variables with more than two response alternatives.

### Respiratory symptoms as risk factors for health care consumption

In a regression model, wheeze and attacks of shortness of breath in the past 12 months and recurrent sputum production increased the risk for health care consumption. Recurrent day- and night-time cough increased the risk for consuming different kinds of evaluated health care resources except from regular outpatient care. Recurrent morning cough was not associated with increased risk for health care consumption (Table 3). In another regression model adjusting for working status, the same associations were identified as in Table 3 except that recurrent morning cough was associated with an increased risk for specialist consultations when adjusting for working status. Table 3 also shows that increasing the number of respiratory symptoms increased the risk for consuming all evaluated health care resources.

**Table 3:** Respiratory symptoms and number of respiratory symptoms^a^ in relation to health care consumption in the general population analyzed by multiple logistic regression analyses expressed as odds ratio (OR) with 95% CI, adjusted for country, sex, age, BMI, smoking status

	Outpatient care during the last year		Regular outpatient care		Specialist consultations		Hospitalisations	
				
Symptom	OR	95% CI	OR	95% CI	OR	95% CI	OR	95% CI
Recurrent morning cough	1.20	0.95–1.52	1.09	0.70–1.70	1.28	0.98–1.67	1.10	0.82–1.48
Recurrent day- and night-time cough	**1.49**	**1.19–1.85**	1.49	0.98–2.28	**1.37**	**1.07–1.75**	**1.39**	**1.06–1.84**
Recurrent sputum production								
Now and then	**1.63**	**1.29–2.05**	**1.88**	**1.16–3.03**	**1.30**	**1.00–1.70**	**1.48**	**1.10–1.98**
Often	**2.10**	**1.51–2.90**	**2.41**	**1.33–4.34**	**1.86**	**1.30–2.66**	**1.65**	**1.09–2.49**
Wheeze in the past 12 months	**3.00**	**2.41–3.72**	**4.72**	**3.01–7.42**	**2.95**	**2.31–3.76**	**2.60**	**1.97–3.44**
Number of symptoms^a^								
0 symptoms	**1**		**1**		**1**		**1**	
1 symptom	**2.89**	**2.06–4.05**	**8.24**	**2.33–29.06**	**2.56**	**1.78–3.68**	**2.34**	**1.55–3.53**
2 symptoms	**5.66**	**4.05–7.91**	**29.97**	**9.10–98.66**	**4.90**	**3.41–7.04**	**3.63**	**2.39–5.50**
3 symptoms	**8.87**	**6.28–12.52**	**35.28**	**10.54–118.05**	**6.10**	**4.15–8.97**	**5.39**	**3.52–8.25**
4 symptoms	**14.12**	**9.82–20.30**	**79.59**	**24.03–263.66**	**13.91**	**9.44–20.50**	**9.13**	**5.87–14.21**
5 symptoms	**28.71**	**18.33–44.96**	**169.32**	**49.75–576.25**	**29.49**	**18.45–47.13**	**16.23**	**9.58–27.49**

Negative answers as reference in each variable.

Significant risk factors are depicted in bold.

a A cumulative symptom score based on the following symptoms: Recurrent morning cough, Recurrent day- and night-time cough, Recurrent sputum production, Wheeze in the past 12 months, Attacks of shortness of breath in the past 12 months. The variable ‘Number of symptoms’ was not analysed in the same multiple regression model as the symptoms.

### Health care consumption among subjects with any respiratory symptoms

Prevalence estimations of health care consumption in each country and among males and females are presented in Table 4. The consumption of health care resources differed between the three countries, both the proportion of subjects who had consulted the health care sector during the past year and also the type of consultations made (Table 4). Subjects in Sweden had significantly more regular visits to outpatient care compared with subjects in Finland. Specialist consultations were significantly more common in Finland compared with in Sweden and in Estonia. A higher proportion of hospitalisations was found in Estonia compared with in Finland and Sweden.

**Table 4:** Comparing health care consumption (%) among subjects with any respiratory symptoms during the past 12 months between countries, in the total study population and by sex

	Country				Males				Females			
			
	Finland	Estonia	Sweden	*p*#	Finland	Estonia	Sweden	*p*#	Finland	Estonia	Sweden	*p*#
Outpatient care during	19.0	18.8	17.2	F/E^ns^	14.8	13.3	14.8	F/E^ns^	22.5	23.8	19.5	F/E^ns^
the past 12 months				F/S^ns^				F/S^ns^				F/S^ns^
				E/S^ns^				E/S^ns^				E/S^ns^
Regular outpatient care	3.2	4.7	6.4	F/E^ns^	2.8	3.6	6.8	F/E^ns^	3.5	5.6	6.1	F/E^ns^
				F/S***				F/S**				F/S^ns^
				E/S^ns^				E/S*				E/S^ns^
Specialist consultations	19.1	9.9	12.6	F/E***	18.0	6.7	10.7	F/E***	20.0	12.7	14.4	F/E**
				F/S***				F/S***				F/S*
				E/S^ns^				E/S*				E/S^ns^
Hospitalisations	8.8	15.0	6.5	F/E***	7.8	14.9	6.4	F/E***	9.7	15.1	6.6	F/E*
				F/S^ns^				F/S^ns^				F/S^ns^
				E/S***				E/S***				E/S***

F=Finland, E=Estonia, S=Sweden, ns=non-significant. *p*>0.05

* *p*<0.05

** *p*<0.01

*** *p*<0.001

# *p*-values based on Chi-square test.

### Risk factors of outpatient care during the past 12 months

In the total sample, Finnish and Estonian nationality, female sex, BMI>25 were associated with an increased risk of outpatient care consumption, while a BMI<20 decreased the risk. However, analyses stratified by country revealed that the risk factor pattern varied between the three countries; in Finland being an ex-smoker increased the risk to consume outpatient care. In Estonia, a BMI between 25 and 30 and in Sweden a BMI >30 increased the risk, and in Estonia a BMI<20 decreased the likelihood to consume outpatient care. In Sweden, subjects not working were more likely to seek outpatient care (Table 5).

**Table 5:** Risk factors associated with health care consumptions assessed as outpatient care during the past 12 months expressed as odds ratio (OR) with 95% CI, analysed by multiple logistic regression analyses in the total study population and stratified by country

	Risk factors for outpatient care during the last 12 months							
	
	All subjects		Finland		Estonia		Sweden	
				
	OR	95% CI	OR	95% CI	OR	95% CI	OR	95% CI
Country								
Sweden	1							
Finland	**1.38**	**1.12–1.72**						
Estonia	**1.30**	**1.04–1.64**						
Sex								
Male	1		1		1		1	
Female	**1.75**	**1.44–2.12**	**1.76**	**1.26–2.45**	**2.16**	**1.46–3.20**	**1.53**	**1.12–2.07**
Age^a^	1.00	0.99–1.01	1.01	1.00–1.03	1.00	0.98–1.01	0.99	0.98–1.00
BMI								
20–25	1				1		1	
<20	**0.49**	**0.28–0.89**	0.48	0.17–1.40	**0.22**	**0.05–0.92**	0.83	0.37–1.88
>25–30	**1.31**	**1.07–1.62**	0.95	0.66–1.36	**1.72**	**1.16–2.55**	1.40	0.99–1.98
>30	**1.53**	**1.18–1.98**	1.29	0.85–1.97	1.50	0.91–2.48	**1.88**	**1.20–2.92**
Smoking status								
Non-smokers	1				1		1	
Ex-smokers	1.24	0.99–1.56	**1.72**	**1.18–2.50**	0.93	0.55–1.57	1.12	0.79–1.61
Current smokers	1.21	0.96–1.50	1.37	0.93–2.03	1.14	0.78–1.72	1.13	0.78–1.66
Working status								
Working/studying	1		1		1		1	
Not working	1.15	0.91–1.45	0.76	0.51–1.14	1.23	0.78–1.92	**1.72**	**1.17–2.52**
Old age pension	1.00	0.70–1.14	0.92	0.51–1.66	0.63	0.33–1.20	1.44	0.79–2.61

a Continuous variable. BMI=body mass index. Significant risk factors are depicted in bold.

### Impact of respiratory symptoms on everyday life among subjects with any respiratory symptoms

**Fig. 1:**
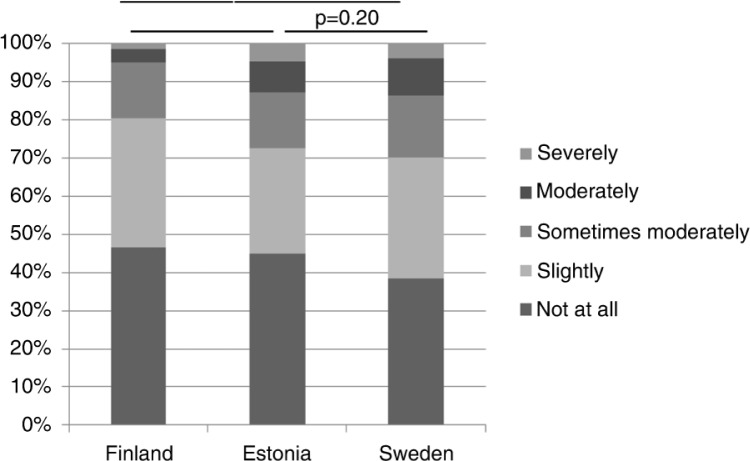
The impact of respiratory symptoms on everyday life among subjects with any respiratory symptoms during the past 12 months based on Mantel–Haenszel test for trends.

**Fig. 2:**
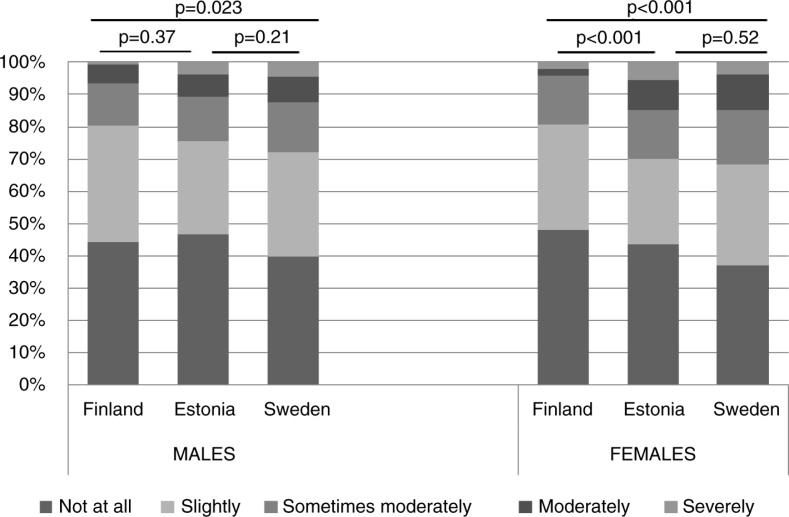
The impact of respiratory symptoms on everyday life among males and females with any respiratory symptoms during the past 12 months based on Mantel–Haenszel test for trends.

Reported impact of respiratory symptoms on everyday life differed between the investigated countries. Among subjects with respiratory symptoms in Sweden and Estonia, a larger proportion was moderately or severely affected by their respiratory symptoms in everyday life when compared with corresponding relationships in Finland (Fig. 1). The same pattern was observed among females (Fig. 2). A difference between males in Finland and Sweden was observed: among Swedish males reporting to be affected by the respiratory symptoms, there was a larger proportion of subjects who was moderately or severely affected by the symptoms compared with Finnish males (Fig. 2). Table 6 shows that each investigated respiratory symptom affected everyday life moderately to severely and that the increasing number of respiratory symptoms increased the risk for being influenced in everyday life.

**Table 6:** Respiratory symptoms and number of respiratory symptoms in relation to experienced impact, moderately to severely, on everyday life analysed by unadjusted logistic regressions and multiple regression models expressed as odds ratio (OR) with 95% CI, adjusted for country, sex, age, BMI, and smoking status

	Unadjusted analyses		Adjusted analyses	
		
	Impact on everyday life^a^		Impact on everyday life^a^	
		
Symptom	OR	95% CI	OR	95% CI
Recurrent morning cough	**3.05**	**2.46–3.78**	**1.59**	**1.21–2.10**
Recurrent day- and night-time cough	**3.12**	**2.51–3.87**	**1.86**	**1.44–2.42**
Recurrent sputum production				
Now and then	**2.04**	**1.59–2.63**	**1.40**	**1.03–1.90**
Often	**4.61**	**3.45–6.15**	**2.28**	**1.57–3.31**
Wheeze in the past 12 months	**3.50**	**2.81–4.37**	**2.09**	**1.61–2.71**
Attacks of shortness of breath in the past 12 months	**4.42**	**3.54–5.51**	**2.89**	**2.24–3.72**
Number of symptoms^b^				
0 symptoms	1		1	
1 symptom	**3.17**	**1.77–5.68**	**3.37**	**1.84–6.16**
2 symptoms	**5.65**	**3.23–9.87**	**6.42**	**3.60–11.45**
3 symptoms	**7.91**	**4.56–13.71**	**8.80**	**4.94–15.68**
4 symptoms	**19.93**	**11.49–34.57**	**23.77**	**13.32–42.41**
5 symptoms	**38.49**	**20.96–70.65**	**41.61**	**21.92–79.01**

Significant risk factors are depicted in bold

a In the unadjusted analyses, the respiratory symptoms were analysed separately one by one and in the adjusted analyses all respiratory symptoms were entered in the same model and adjusted for country, sex, age, BMI, and smoking status.

b Number of symptoms was not analysed in the same multiple regression model as the symptoms. Negative answers as reference in each variable.

## Discussion

In this population-based study, the prevalence of any respiratory symptom was significantly higher in Estonia and Finland compared with in Sweden, with a similar pattern among males and females. An increasing number of respiratory symptoms were associated with an increased risk for health care consumption; outpatient care during the last year, regular outpatient care, and specialist consultations as well as hospitalisations. Among subjects with respiratory symptoms, the proportion reporting outpatient care during the past year was fairly similar in the three countries. However, there were differences regarding pattern of health care consumption among those with any respiratory symptoms: regular outpatient visits were most common in Sweden, specialist consultations were most common in Finland, and the proportion of hospitalisations was highest in Estonia. Also, the reported impact of respiratory symptoms on everyday life differed between the three countries. Although respiratory symptoms were more prevalent in Finland, the symptoms seemed to affect everyday life more among the participants in Estonia and Sweden. The experienced impact on everyday life increased by the number of respiratory symptoms.

The prevalence of any respiratory symptoms in the current study was higher than previously found in an US population (4). However, when considering the prevalence of a single symptom as for instance wheeze, the prevalence in the current study was rather similar to the prevalence of around 23% in Australian (3) and Norwegian populations (5) but much higher than the prevalence of around 13% found in an US population (4). One explanation for these discrepancies between studies regarding prevalence of respiratory symptoms may be that the definitions of any respiratory symptoms and single respiratory symptoms differ between studies. Another explanation could also be due to differences related to sampling or study area. In our study, the prevalence of any respiratory symptom was on a similar level in Finland and Estonia but higher compared with in Sweden. One possible explanation for this finding could be differences in smoking habits; current smoking is a well-known risk factor for respiratory symptoms (12), and in a previous report from northern Sweden, a trend of decreasing respiratory symptoms over time was parallel to a lower prevalence of smoking (9). In our study, the prevalence of current smokers in Finland was lower than in Estonia but the proportion of ex-smokers was higher in Finland than in Estonia, which indicates that smoking could be one explanatory factor for the higher prevalence of respiratory symptoms in these both countries. Contradictory to this possible explanation is the fact that the prevalence of ex-smokers in Sweden was similar to the prevalence in Finland but the prevalence of any respiratory symptoms was significantly lower in Sweden. The results indicate that other factors, besides smoking, may contribute to the observed difference related to study area, for example, environmental factors and/or occupational exposure (3, 13–16). However, such data were not included in the current study.

The health care consumption differed between the three countries, and this could of course be due to different structures in the health care systems. Despite significant efforts to develop the primary care sector and national programmes for respiratory conditions (28–30), nearly one in five of the Finnish subjects with respiratory symptoms had consulted a specialist. The high proportion of specialist consultation may also depend on local organisation of occupational health services and availability of private health care providers in Finland. The timing of the study around the turn of the millennium may be too early to assess the impact of the referred national programmes, and further follow-up studies are warranted. In Sweden, there is a virtual scarcity of private sector services in respiratory medicine, and most specialist consultations, both outpatient and in-patient services, take place within the domain of public health care. In general, most patients with respiratory problems in Sweden will first contact their primary health care centre. Also in Estonia, the timing of the study might explain the higher level of hospitalisations, and more outpatient-oriented services having been developed during the recent years. The differences in the proportions of hospitalisations between countries are probably related to different health care structures and availability of services and local arrangements.

It has previously been reported that there are different cultures in diagnosing obstructive lung diseases in the three investigated countries (31), but the focus of the present study was respiratory symptoms and not established diagnoses of respiratory diseases. We hypothesised that it is primarily the experience of respiratory symptoms and not a given diagnosis that affects everyday life and also influences health care consumption. The study confirmed our hypothesis; an increasing number of respiratory symptoms were associated with an increased risk for health care consumption, namely outpatient-, specialist-, and hospital care, and this was more pronounced among females. Most research in the Nordic countries has focused on health care consumption in relation to obstructive lung diseases (17–21), but our results indicate that respiratory symptoms as well as number of symptoms, irrespective of diagnosis, contribute to a considerable health care consumption and consequently high health economic costs in Finland, Estonia, and Sweden. Interestingly, in a recently published study from another part of the world, namely India, the most common cause for seeking primary health care was respiratory symptoms (32).

Furthermore, in the current study, subjects with BMI over 30 were more likely to seek outpatient care due to respiratory symptoms. This finding is in line with previous observations that higher BMI in general, without specific focus on respiratory conditions, is associated with higher health care expenditure (33). It has also been reported that females in general have a higher health care consumption than males (34, 35), and in our study females were more inclined to seek outpatient care despite rather similar prevalence of respiratory symptoms as among males. This may indicate that males are more hesitant to seek health care for their respiratory symptoms, which in a long-term perspective could have negative consequences for their respiratory health. Sex differences in health care utilisation among subjects with respiratory symptoms need to be further evaluated.

Importantly, respiratory symptoms are not only to be regarded as a societal burden but also a burden for the individual as well. In our study, a majority of subjects experienced that their everyday life was influenced in varying degrees by respiratory symptoms, symptoms known to have a negative impact on HRQL (4, 22). In particular, respiratory symptoms such as cough, wheeze, phlegm, and breathlessness contribute not only to poorer physical HRQL but also, though to a lesser degree, poorer mental HRQL (22). It is natural to assume that the subjects in the current study who experienced their everyday life being affected by an increasing number of symptoms also had a deteriorated HRQL. It is indeed a weakness that our study did not include any validated assessment of HRQL; however, the results indicate that further research focusing on respiratory symptoms in relation to impact on everyday life and HRQL is important.

The strengths of the current study were that random samples from large-scale population studies were examined, and the results can be regarded as representative of the populations in the investigated areas in Finland, Estonia, and Sweden. Furthermore, a well-validated questionnaire was used during the structured interview. Although data were collected a few years ago and the structure of the health care systems are under constant change, the observed relationships are important to consider. The results highlight the need for future studies, preferably on a national level due to the observed differences between countries, to evaluate changes regarding the prevalence of respiratory symptoms and the related health care consumption. Such studies may help to predict the need of medical care resources for respiratory problems. The data on impact of respiratory symptoms on everyday life are based on a very simple question with five response options, which are easy to use when meeting the patients. However, it could be further validated in relation to established questionnaires assessing both generic and disease-specific (assessing respiratory conditions) HRQL.

In conclusion, this population-based study shows that the prevalence of any respiratory symptoms was high and consumption of health care resources due to respiratory symptoms was common in the three countries, even though there were some differences between the investigated countries. Our finding indicates that respiratory symptoms contribute to a considerable health burden as well as economic burden for the society and also have a considerable impact on the everyday life of an individual.

## References

[1] Lundbäck B, Stjernberg N, Nyström L, Forsberg B, Lindström M, Lundbäck K (1994). Epidemiology of respiratory symptoms, lung function and important determinants. Tuberc Lung Dis.

[2] Frostad A, Soyseth V, Haldorsena T, Andersena A, Gulsvik A (2007). Respiratory symptoms and long-term cardiovascular mortality. Respir Med.

[3] James AL, Knuiman MW, Divitini ML, Hui J, Hunter ML, Mulrennan SA (2013). Risk factors for respiratory symptoms in adults: the Busselton Health Study. Respirology.

[4] Wheaton AG, Ford ES, Thompson WW, Greenlund KJ, Presley-Cantrell LR, Croft JB (2013). Pulmonary function, chronic respiratory symptoms, and health-related quality of life among adults in the United States – National Health and Nutrition Examination Survey 2007–2010. BMC Public Health.

[5] Voll-Aanerud M, Eagana TM, Wentzel-Larsen T, Gulsvik A, Bakke PS (2008). Respiratory symptoms, COPD severity, and health related quality of life in a general population sample. Respir Med.

[6] Grønseth R, Vollmer WM, Hardie JA, Ólafsdóttir IS, Lamprecht B, Buist AS (2014). Predictors of dyspnoea prevalence: results from the BOLD study. Eur Respir J.

[7] Ekerljung L, Andersson Å, Sundblad B-M, Rönmark E, Larsson K, Ahlstedt S (2010). Has the increase in the prevalence of asthma and respiratory symptoms reached a plateau in Stockholm, Sweden?. Int J Tuberc Lung Dis.

[8] Kainu A, Pallasaho P, Piirilä P, Lindqvist A, Sovijärvi A, PIetinalho P (2013). Increase in prevalence of physician-diagnosed asthma in Helsinki during the Finnish Asthma Programme: improved recognition of asthma in primary care?. Prim Care Respir J.

[9] Backman H, Hedman L, Jansson SA, Lindberg A, Lundbäck B, Rönmark E (2014). Prevalence trends in respiratory symptoms and asthma in relation to smoking – two cross-sectional studies ten years apart among adults in northern Sweden. World Allergy Organ J.

[10] Viegi G, Pedreschi M, Baldacci S, Chiaffi L, Pistelli F, Modena P (1999). Prevalence rates of respiratory symptoms and diseases in general population samples of North and Central Italy. Int J Tuberc Lung Dis.

[11] Lindström M, Kotaniemi J, Jönsson E, Lundbäck B (2001). Smoking, respiratory symptoms and diseases. Chest.

[12] Pallasaho P, Lindström M, Põlluste J, Loit H-M, Sovijärvi A, Lundbäck B (2004). Low socio-economic status is a risk factor for respiratory symptoms: a comparison between Finland, Sweden and Estonia. Int J Tuberc Lung Dis.

[13] Kurmi OP, Semple S, Devereux GS, Gaihre S, Lam KBH, Sadhra S (2014). The effect of exposure to biomass smoke on respiratory symptoms in adult rural and urban Nepalese populations. Environ Health.

[14] Kim JL, Torén K, Lohman S, Ekerljung L, Lötvall J, Lundbäck B (2013). Respiratory symptoms and respiratory-related absence from work among health care workers in Sweden. J Asthma.

[15] Khan AW, Moshammer HM, Kundi M (2015). Industrial hygiene, occupational safety and respiratory symptoms in the Pakistani cotton industry. BMJ Open.

[16] Ghosh T, Gangopadhyay S, Das B (2014). Prevalence of respiratory symptoms and disorders among rice mill workers in India. Environ Health Prev Med.

[17] Jansson SA, Backman H, Stenling A, Lindberg A, Rönmark E, Lundbäck B (2013). Health economic costs of COPD in Sweden by disease severity – has it changed during a ten years period. Respir Med.

[18] Jansson SA, Andersson F, Borg S, Ericsson Å, Jönsson E, Lundbäck B (2002). Costs of COPD in Sweden according to disease severity. Chest.

[19] Nielsen R, Johannessen A, Benediktsdottir B, Gislason T, Buist AS, Gulsvik A (2009). Present and future costs of COPD in Iceland and Norway: results from the BOLD study. Eur Respir J.

[20] Bilde L, Svenning AR, Dollerup J, Bække Borgeskov H, Lange P (2007). The cost of treating patients with COPD in Denmark – a population study of COPD patients compared with non-COPD controls. Respir Med.

[21] Jansson SA, Rönmark E, Forsberg B, Löfgren C, Lindberg A, Lundbäck B (2007). The economic consequences of asthma among adults in Sweden. Respir Med.

[22] Voll-Aanerud M, Eagan TM, Plana E, Omenaas ER, Bakke PS, Svanes C (2010). Respiratory symptoms in adults are related to impaired quality of life, regardless of asthma and COPD: results from the European community respiratory health survey. Health Qual Life Outcomes.

[23] Lundbäck B, Nyström L, Rosenhall L, Stjernberg N (1991). Obstructive lung disease in northern Sweden: respiratory symptoms assessed in a postal survey. Eur Respir J.

[24] Medical Research Council Committee on the Aetiology of Chronic Bronchitis (1665). Standardized questionnaires on respiratory symptoms. Br Med J. 1960.

[25] Pallasaho P, Lundbäck B, Meren M, Kiviloog J, Loit HM, Larsson K (2002). Prevalence and risk factors for asthma and chronic bronchitis in the capitals Helsinki, Stockholm, and Tallinn. Respir Med.

[26] Meren M, Raukas-Kivioja A, Jannus-Pruljan L, Loit HM, Rönmark E, Lundbäck B (2005). Low prevalence of asthma in westernizing countries-myth or reality? Prevalence of asthma in Estonia – a report from the ‘FinEsS’ study. J Asthma.

[27] Jannus-Prulian L, Meren M, Polluste J, Loit H-M, Kiviloog J, Baburin A (2004). Postal survey on asthma, chronic bronchitis and respiratory symptoms among adult Estonians and non-Estonians (FinEsS-study). Eur J Public Health.

[28] Kinnula VL, Vasankari T, Kontula E, Sovijarvi A, Saynajakangas O, Pietinalho A (2011). The 10-year COPD Programme in Finland: effects on quality of diagnosis, smoking, prevalence, hospital admissions and mortality. Prim Care Respir J.

[29] Haahtela T, Laitinen LA (1996). Asthma programme in Finland 1994–2004. Report of a working group. Clin Exp Allergy.

[30] Laitinen LA, Koskela K (1999). Chronic bronchitis and chronic obstructive pulmonary disease: Finnish National Guidelines for Prevention and Treatment 1998–2007. Respir Med.

[31] Pallasaho P, Meren M, Raukas-Kivioja A, Rönmark E (2005). Different labelling of obstructive airway diseases in Estonia, Finland, and Sweden. Eur J Epidemiol.

[32] Salvi S, Apte K, Madas S, Barne M, Chhowala S, Sethi T (2015). Symptoms and medical conditions in 204 912 patients visiting primary health-care practitioners in India: a 1-day point prevalence study (the POSEIDON study). Lancet Global Health.

[33] Buchmueller TC, Johar M (2015). Obesity and health expenditures: evidence from Australia. Econ Hum Biol.

[34] Osika-Friberg I, Krantz G, Määttä S, Järbrink K (2015). Sex differences in health care consumption in Sweden: a registerbased cross-sectional study. Scand J Public Health.

[35] Wang Y, Hunt K, Nazareth I, Freemantle N, Petersen I (2013). Do men consult less than women? An analysis of routinely collected UK general practice data. BMJ Open.

